# Phosphatidylserine Outer Layer Translocation Is Implicated in IL-10 Secretion by Human Regulatory B Cells

**DOI:** 10.1371/journal.pone.0169755

**Published:** 2017-01-10

**Authors:** Rachel Audo, Charlotte Hua, Michael Hahne, Bernard Combe, Jacques Morel, Claire I. Daien

**Affiliations:** 1 Department of Rheumatology, University Hospital Center of Montpellier, Montpellier, France; 2 TNF-alpha family laboratory, UMR5535, IGMM, CNRS, Montpellier, France; Monash University, AUSTRALIA

## Abstract

B cells can have a regulatory role, mainly mediated by interleukin 10 (IL-10). IL-10 producing B cells (B10 cells) cells remain to be better characterized. Annexin V binds phosphatidylserine (PS), which is externalized during apoptosis. Previous works suggested that B10 cells are apoptotic cells since they bind Annexin V. Others showed that Annexin V binding could also be expressed on viable B cells. We aimed to explore if PS exposure can be a marker of B10 cells and if PS exposure has a functional role on B cell IL-10 production in healthy subjects. We found that B10 cells were significantly more often Annexin V^+^ than IL-10 non-producing B cells. After CpG activation, Annexin V^+^ B cells differentiated more often into B10 cells than Annexin V^neg^ B cells. Cell death and early apoptosis were similar between Annexin V^+^ and Annexin V^neg^ B cells. PS blockage, using biotinylated AnV and glyburide, decreased B10 cell differentiation. This study showed that B10 cells have an increased PS exposure independently of any apoptotic state. B cells exposing PS differentiate more into B10 cells whereas PS blockage inhibits B10 cells generation. These results strongly suggest a link between PS exposure and B10 cells.

## Introduction

B cells have a promoting role in auto-immune diseases, which is mediated by autoantibody production, antigen presenting functions and pro-inflammatory cytokine secretion. However, B cells can also have a negative regulatory role. These so-called regulatory B cells were originally identified in relevant inflammatory mouse models, including arthritis, by their ability to improve already established disease in transfer experiments [1,2]. The regulatory B cells are mainly characterized by their secretion of interleukin 10 (IL-10) and so are often called B10 cells. We recently showed decreased B10-cell count in rheumatoid arthritis (RA) patients [3]. On the other hand, several studies have implicated B10 cells as immunosuppressive drivers promoting malignancy progression [4]. IL-10 is overexpressed in human chronic lymphocytic leukemia (CLL) and human malignant CLL cells can produce autocrine IL-10 [5,6]. IL-10 is crucial for the development of malignant B clones in CLL NZB mice models [7,8]. IL-10 inhibits B cell apoptosis in advanced stages of CLL [9–11]. Therefore, promoting B10 cells in auto-immune diseases and inhibiting B cell IL-10 production in CLL are promising therapeutic tools, requiring a better comprehension of B cell IL-10 production regulation.

Annexin V (AnV) is a member of a large family of Ca2+ and phospholipid binding proteins [12]. AnV has high affinity for negatively charged phospholipids, especially phosphatidylserine (PS). The plasma membrane of a healthy cell typically exhibits an asymmetric distribution of its major phospholipids. Virtually all the PS resides on the inner leaflet of the plasma membrane. During the early stages of apoptosis, cells lose their membrane phospholipid asymmetry and expose PS on the outer leaflet of the plasma membrane, making of AnV a known marker for early apoptosis. However, it has been reported that PS could also be externalized on viable B cells during process other than apoptosis and could play a role in cell signaling [12,13].

Relationships between regulatory B cells and apoptosis have been previously suggested. Two studies showed that regulatory B cells (defined as IL-10 producing CD19^+^CD5^+^ and CD19^+^CD5^+^Foxp3^+^ B cells respectively) had a strong AnV staining, interpreted as apoptosis [14,15]. We hypothesized that PS exposure could be linked to B cell activation in regulatory B cells instead of apoptosis. We therefore studied PS exposure and its function in human B10 cells of healthy subjects.

## Materials and Methods

### Subjects

Healthy subjects were either blood donors or patients seen in rheumatology department (CHU Montpellier) for mild osteoarthritis, vertebral discopathy or other mechanical pain free of any general pathology or infection. All patients and blood donors gave written informed consent to participate in the study as approved by the Medical Ethics Committee of Nimes hospital, France (n° 2012-A00592-41).

### Cell isolation and cell culture

Blood was collected into tubes containing EDTA. PBMCs were isolated from whole blood using Ficoll-Paque Plus (GE Healthcare, Aulnay-sous-bois, France).

Total PBMCs were cultured in filtered RPMI 1640 (LifeTechnologies, Saint-Aubin, France) with 10% fetal calf serum (FCS) with penicillin (100 UI/ml)/ streptomycin (100 UI/ml) for 24 or 72 hr on 96-well plates at 1.5 x 10^6^ cells/ml and 200 μl per well.

### Cell activation for assessing IL-10 producing B cells and flow cytometry

PBMCs were activated overnight with 10μg/ml CpG (Toll-like receptor 9 ligand, ODN 2006; InvivoGen, Toulouse, France) as previously described [16]. Phorbol 12-myristate 13-acetate (0.4μg/ml) and ionomycine (0.5μg/ml) were added for the last 4 hr of culture and brefeldin A (10μg/ml) for the last 2 hr of culture.

Activated cells were stained for cell surface markers: B cells were phenotyped by use of Horizon V450-conjugated anti-CD19 (BD Horizon, Le Pont-de-Claix, France) and fluorescein isothiocyanate (FITC)-conjugated AnnexineV or APC-conjugated Annexine V (Roche Life Science, Meylan, France) in Annexin V Binding buffer (0.1M HEPES, pH 7.4, 1.5 M NaCl, 25mM CaCl_2_). Exclusion of dead cells was performed using Fixable Viability Dye eFluor 506. Cells were then permeabilized with Cytofix/Cytoperm buffer for 30 min on ice (BD Biosciences, Le pont-de-Claix, France) and 1X Perm/wash Buffer (BD Biosciences) 10 min on ice before staining with allophycocyanin (APC)-conjugated anti-IL-10 antibodies (BD Pharmingen, Le Pont-de-Claix, France) or APC-conjugated isotype control (BD Pharmingen) diluted in 1X Perm/wash buffer. Cells were then analyzed by use of FACSCanto II (BD Bioscience) at the MRIO facility. Gating strategies for flow cytometry analysis were performed using Flowjo software 6.3 (Treestar, Ashland, OR). First, PBMCs were gated based on forward scatter (FSC) and side scatter (SSC), followed by exclusion of doublets and dead cells exclusion. Then, B cells were gated according to CD19 and AnV positive expression. Last, B10 cells were gated according to IL-10 positive expression (S1 Fig).

To assess AnV staining on unactivated B cells known to be regulatory B cells precursors (i.e CD19+CD24^hi^CD38^hi^, CD19+CD5+ and CD19+CD24^hi^CD27^hi^), PBMCs were stained 20 minutes on ice with FITC-conjugated AnnexineV (Roche Life Science) and for B cells surface markers: APC-conjugated anti-CD19 (BD Pharmingen), Phycoerythrin-cyanine 7 (PeCy7)-conjugated anti-CD27 (BD Pharmingen), PE-conjugated anti-CD24 (BD Pharmingen), APC-Hillite7 (ACP-H7)-conjugated anti-CD38 (BD Biosciences), Peridinin-chlorophyll-cyanine 5.5 (PerCp-Cy5.5)-conjugated anti-CD5 (BioLegend, London, United Kingdom) in Annexin V Binding buffer. Cells were then washed and resupended in Annexin V Binding buffer containing DAPI before FACS analysis.

### Differentiation of annexin V positive B cells into B10 cells

PBMCs were isolated from blood samples of 6 healthy donors and stained for CD19 and AnV expression, as described previously. CD19+ B cells were then sorted according to their AnV staining using the cell sorter FACS-ARIA III (BD Biosciences), in order to obtain 2 populations: the B cells with positive AnV staining (AnV^+^) and the B cells with a negative AnV staining (AnV^neg^). Dead cells were excluded from sorting using DAPI staining. These 2 B-cell populations were then activated during 24 hr according to the standard protocol described earlier with a slight modification: because the cell culture contained B cells only, CD40-Ligand coated plates were used (1ug/ml). IL-10 secretion or TNF-α, IL-6 and GM-CSF secretion were then assessed using flow cytometry. For TNF-α, phycoerythrin (PE)-conjugated anti-TNFα (e-Biosciences, Paris, France) or with PE-conjugated isotype control (e-Biosciences) were used. For IL-6 and GM-CSF, anti- PECy7-conjugated anti-CD19 (BD Horizon, Le Pont-de-Claix, France) and APC-conjugated AnnexineV (BD Pharmingen), FITC-conjugated anti-IL-6 or PE-conjugated anti GM-CSF (BD Pharmingen) were used.

### Apoptosis and annexin V positive B cells

To show that AnV+ B cells are not only apoptotic cells, several methods were employed. We first used DAPI (Sigma-Aldrich, Saint-Quentin, France) on B cells sorted by FACSARIA according to their AnV staining after 72 hr of culture, to assess the numbers of dead cells. We also used DiOC6 (LifeTechnologies) on B cells sorted by FACSARIA according to their AnV staining, to assess the cells in early apoptosis. Briefly, cells were incubated with DiOC6(3) diluted at 40nM in culture media (1M°cells/ml of dye) protected from light during 30 min at 37°C.

We also used propidium iodide (PI) (LifeTechnologies) to analyze by flow cytometry cell cycle in B cells according to PS exposure level. This allow quantification of the cells in the subG1 phase, corresponding to an apoptotic state. Briefly, PBMC, stained as previously described using anti-19 antibodies and annexin V for extracellular staining, were fixed in Cytofix/Cytoperm buffer for 30 min on ice (BD Biosciences, Le pont-de-Claix, France) and incubated 30min at room temperature in 1X Perm/wash Buffer, BD Biosciences) with addition of PI (50μg/ml) and RNAse A (100μg/ml).

### Impact of PS blockage on IL-10 secretion

To test the impact of PS blockage on IL-10 secretion, we incubated PBMCs with either biotinylated annexin V (BD pharmingen) or glyburide (Invivogen) which is an inhibitor of phospholipid translocase interfering with PS [17,18]. For biotinylated annexin blocking, we used protocol described in Dillon et al. with some modification due to culture of the cells [13]. Briefly, 2.10^6^ of PBMCs were washed, pre-incubated in annexin binding buffer containing 5 μl of biotinylated Annexin V (BD pharmingen), washed and further incubated with streptavidine. Cells were then washed and resuspended in RPMI. Concerning blocking using glyburide, 2.10^6^ of PBMCs were preincubated 30 min with glyburide (Invivogen) at 200 μM or with vehicle control. Then, we stimulated the cells according to the protocol described earlier in order to generate IL-10 secreting B cells.

### Statistical analysis

Data are expressed as median (interquartile range 25–75 [IQR]). Comparisons of the different subpopulations involved Wilcoxon’s matched pairs signed rank test. Statistical analysis and graphs involved use of GraphPad Prism 6.0. P<0.05 was considered statistically significant.

## Results

### Phosphatidylserine exposure is increased on B10 cells

**Fig 1:**
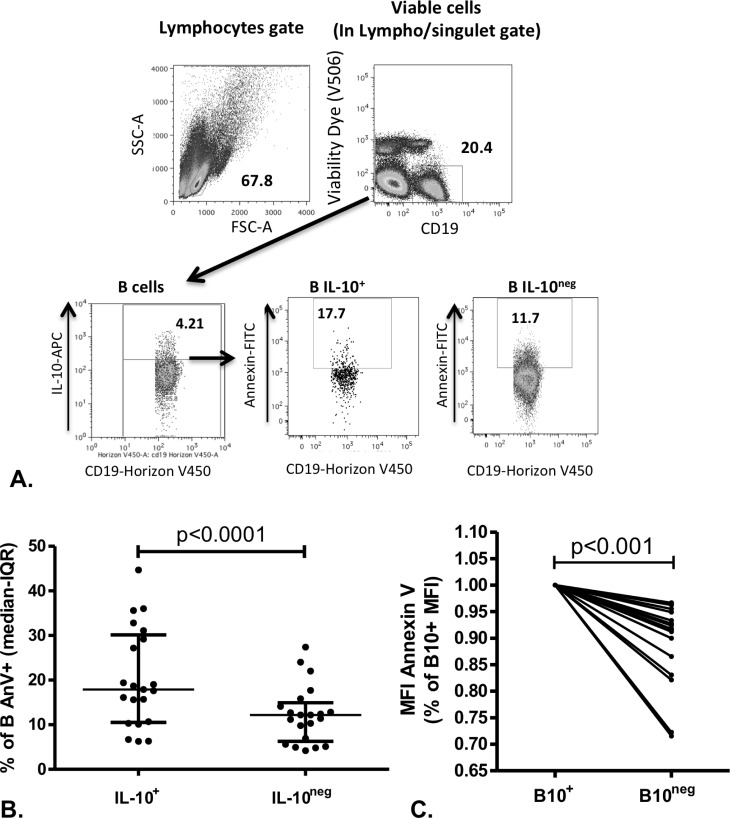
Annexin V binding is increased in IL-10 producing B cells (B10 cells) compared to IL-10 non-producing B cells and to TNFα producing B cells. PBMCs were stimulated for 24 hr with CpG/ionomycin/PMA and BFA as described previously and analyzed by FACS for annexin V (AnV) binding and IL-10 and TNFα production in 21 subjects. Cytometry gating is shown in A. Results are presented in percentage of positive cells in B and median of fluorescence (MFI) in C. Wilcoxon’s matched pairs signed rank tests were used. Top of the bar represents the median, and whiskers are IQR 25–75.

B10 cells were generated from Peripheral blood mononuclear cells (PBMCs) of 21 healthy subjects using the protocol for B10 cell assessment. Briefly, PBMCs were cultured for 24 hours with CpG and 4 hours with ionomycine/PMA and assessed for extracellular CD-19 and intracellular IL-10. Those cells were also stained with annexin V (AnV) in order to detect phosphatidylserine (PS) exposure on B10 cell surface. AnV+B10 cells were assessed using flow cytometry as described in Methods (Fig 1A and S1 Fig). The median (IQR) frequency of AnV+B cells was 17.90 (10.20–28.8)% for IL-10^+^TNFα^neg^ B cells, and 11.20 (5.67–13.45)% for IL-10^neg^ B cells (p<0.0001, n = 21) (Fig 1B). Of note, similar results were found when using APC-conjugated AnV (S2 Fig). AnV Median of fluorescent intensity (MFI) was also higher in IL-10 producing B cells compared to IL-10^neg^ B cells (Ann V MFI in B10neg cells: 91.7 (84.8–95.2)% of AnnV MFI in B10+ cells, p<0.0001)(Fig 1C).

### Phosphatidylserine exposure is increased on B10 cell precursors

**Fig 2:**
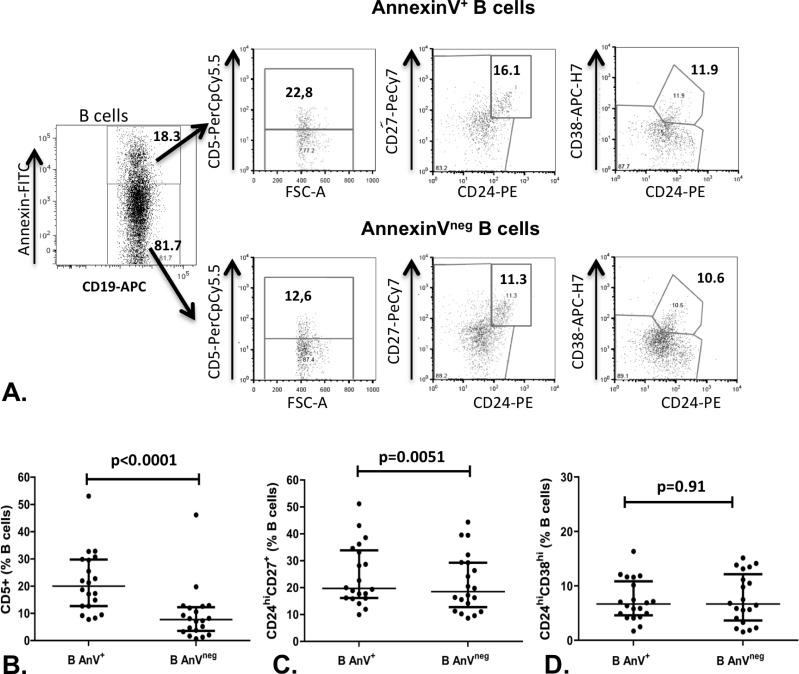
Annexin V binding is increased among CD5+ and CD24hiCD27+ B10 precursors. A representative plot is presented (A). PBMCs from 12 subjects were analyzed by FACS for B10 precursors phenotype (i.e CD19+CD5+ (B), CD19+CD24^hi^CD27^+^ (C) and CD19+CD24^hi^CD38^hi^, (D)), and for annexin V binding. Wilcoxon’s matched pairs signed rank tests were used. Data are median (IQR 25–75).

PBMCs from 20 healthy subjects were stained with AnV and with different surface antibodies in order to identify three B cell subpopulations previously established to be B10 cells precursors: CD19^+^CD5^+^, CD19^+^CD24^hi^CD27^+^ and CD19^+^CD24^hi^CD38^hi^ (example of gating Fig 2A)[19–21]. The expression of CD5 was significantly more frequent on AnV+ than on AnV^neg^ B cells (median [IQR25-75]: 20.00 [12.70–29.75]% among AnV+ B cells and 7.72 [3.55–12.30]% among AnV^neg^ B cells; p<0.0001)(Fig 2B). MFI of Annexin FITC fluorescence was significantly higher in CD5^+^ B cells compared to CD5^neg^ B cells (median (IQR) fold increase: 1.20 (1.02–1.90), p = 0.005, data not shown). Similarly, though less pronounced, AnV+ B cells were more frequently CD19^+^CD24^hi^CD27^+^ than AnV^neg^ B cells (19.70 [16.15–33.90]% of AnV+B cells and 18.50 [12.75–29.35]% of AnV^neg^ B cells; p = 0.005)(Fig 2C). In this subset of cells, intensity of Annexin-FITC fluorescence was significantly higher in CD19^+^CD24^hi^CD27^+^ B cells compared to CD19^+^CD24^neg^ B cells (median (IQR) fold increase: 1.15 (1.09–1.24), p = 0.037, n = 20, Data not shown). In contrast, CD19+CD24^hi^CD38^hi^ B cells were unaltered in AnV+ and AnV^neg^ B cells (6.66 [4.61–10.90]% vs 6.65.85 [3.69–12.20]%, respectively; p = 0.91)(Fig 2D).

### B cells exposing PS differentiate more frequently into B10 cells than other B cells

**Fig 3:**
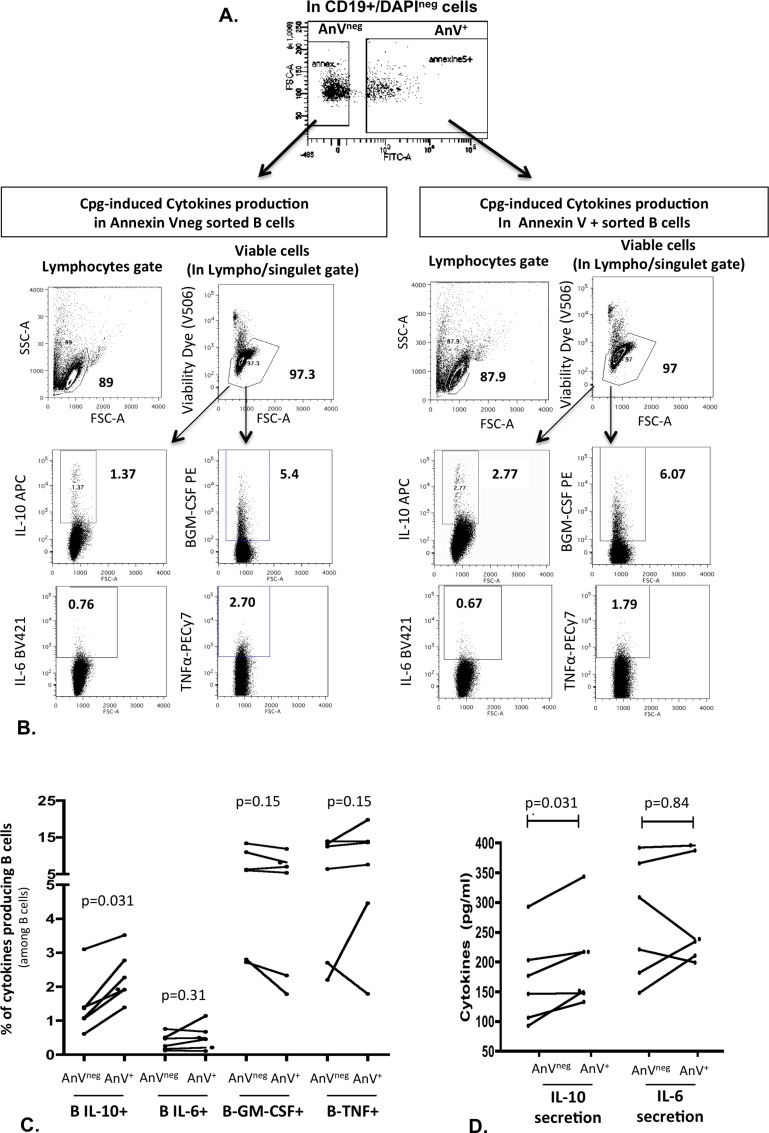
Annexin V positive B cells differentiate more frequently into B10 cells than Annexin V negative B cells. PBMCs from 6 healthy subjects were sorted by flow cytometry (FACSARIA) according to annexin V (AnV) staining: AnV positive (AnV^+^) and AnV negative B cells (AnV^neg^) after exclusion of dead cells (DAPI^+^)(A). Each B cell subpopulation was then stimulated for B10 generation and analyzed by flow cytometry as previously described (B). (C) shows the % of cytokine-positive cells (IL-10, IL-6, GM-CSF and TNF-a) in AnV^+^ and AnV AnV^neg^ sorted cells. (D) shows IL-10 and IL-6 concentrations assessed by ELISA in supernatant of AnV^+^ and AnV AnV^neg^ sorted cells.

To test whether PS+ B cells are prone to develop into B10 cells, we sorted B cells from PBMCs of 6 healthy subjects according to phosphatidylserine exposure: B cells with Annexin V binding (AnV^+^) and B cells with no Annexin V binding (AnV^neg^)(Fig 3A and 3B). After 24 hr of stimulation, the median (IQR25-75) frequency of B10 cells was higher in AnV^+^ purified B cells (2.10 [1.78–2.96] vs 1.22 [0.95–1.82]); p = 0.03)(Fig 3C). Of note, CpG stimulation did not appear to change the AnV staining of B cells (S3 Fig). We also evaluated ability of Annexin V positive sorted B cells to differentiate into IL-6, GM-CSF and TNF-α. In contrast to IL-10, AnV^+^ cells and AnV^neg^ cells expressed similarly IL-6, GM-CSF and TNF-α (Fig 3D). Ability of Annexin V positive sorted B cells to differentiate into B10 cells was also confirmed by ELISA as IL-10 median concentration was higher in the supernatant of AnV^+^ cells than in AnV^neg^ cells (Fig 3C). Conversely, there was no difference of IL-6 production (Fig 3C).

### B cells exposing PS are not apoptotic cells

**Fig 4:**
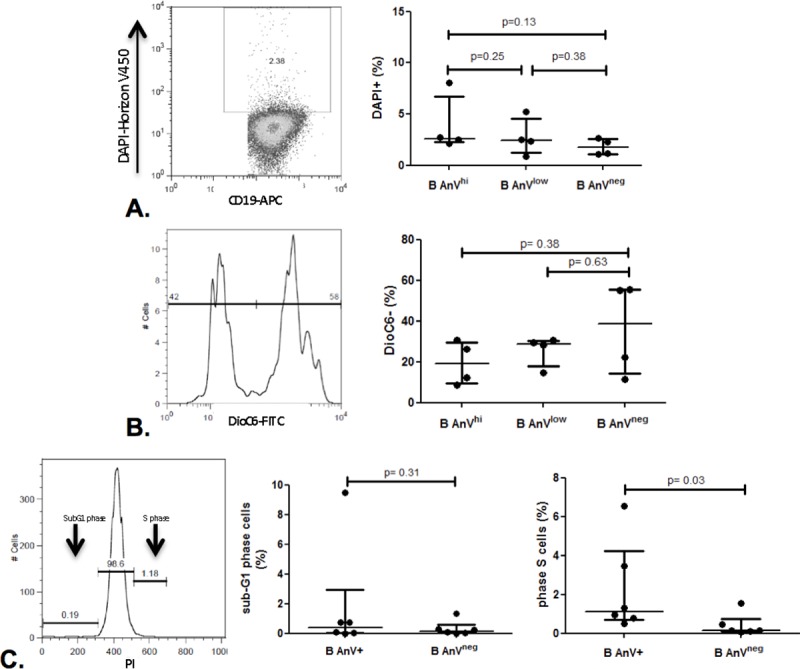
Annexin V positive B cells are not apoptotic cells. PBMCs were stained with APC anti-CD19 and FITC conjugated Annexin V, sorted by FACSARIA according to annexin V (AnV) staining: high AnV B cells (AnV^hi^); low AnV B cells (AnV^low^) and AnV negative B cells (AnV^neg^). Cell death was analyzed at 72 hr using DAPI staining for necrosis (A) and DIOC6 (B) for mitochondrial apoptosis (n = 4). Additionally, cell cycle was analyzed using propidium iodide (PI) staining at 72 hr (C), showing the proportion of cells in the subG1 phase (apoptotic cells) and in the S phase (proliferating cells) in AnV+ (AnV^hi^ and AnV^low^) and AnV^neg^ B cells (n = 5). Data are median (IQR25-75).

After cell sorting to retrieve subpopulations according to PS exposure, AnV^hi^, AnV^low^ and AnV^neg^ B cells were cultured and their viability was assessed using a DAPI staining after 72 hr (Fig 4A). There was a slight but not significant increase of cell death in AnV^hi^ B cells compared to AnV^neg^ and AnV^low^ B cells (p = 0.13 and p = 0.38, respectively; n = 4) but cell death rate was quite low in the three populations (<3%). In order to evaluate early apoptosis, these 3 sub-populations were also stained with DiOC6 (Fig 4B). The percentages of apoptotic cells (DiOC6 -) in AnV^hi^ and AnV^low^ B cells were similar than in AnV^neg^ B cells (p = 0.38 and p = 0.63 respectively; n = 4). Analysis with Live/ Dead staining confirmed that IL-10+ B cells had similar death rates than IL-10^neg^ B cells (data not shown).

We also examined the cellular cycle of AnV^hi^, AnV^low^ and AnV^neg^ non sorted B cells (Fig 4C). The median |IQR25-75] proportion of cells in the subG1 phase (apoptotic cells) was not different in AnV^Hi^ B cells compared to AnV^low^ and AnV^neg^B cells (0.38 [0.15–0.84]% vs 0.0 [0.0–0.34]% and 0.05[0.0–0.28]%, respectively; p = 0.13 for both comparisons; n = 5). Notably, the proportions of cells in the S phase (proliferating cells) were higher although not significantly in AnV^hi^ B cells (5.57 [1.19–8.11]%) and AnV^Low^ B cells (0.59 [0.35–0.93]%) compared to AnV^neg^ B cells(0.13 [0.07–0.31]%); p = 0.06 for both comparisons; n = 5).

### Phosphatidylserine blockage decreases B10 cell differentiation.

**Fig 5:**
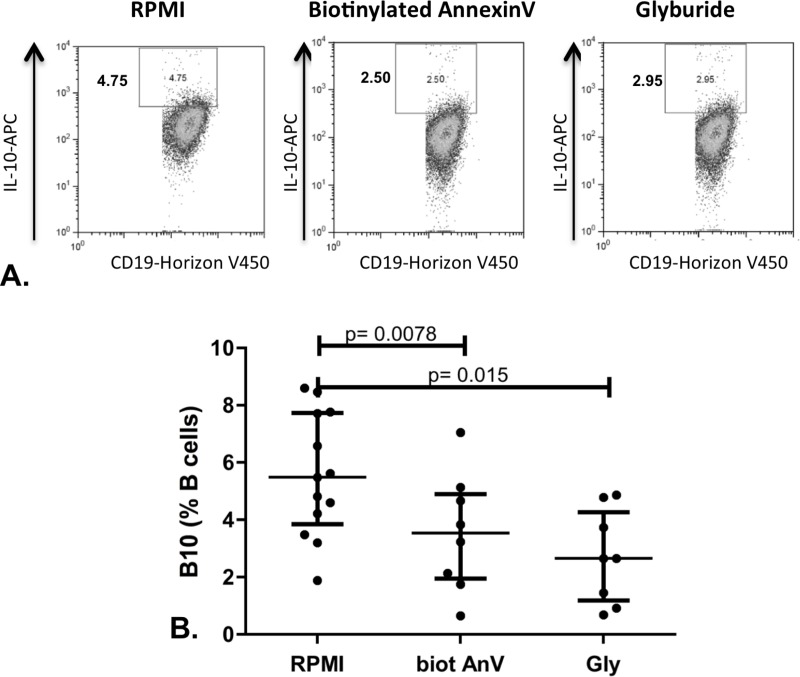
Phosphatidylserine blockage alters B10 cell differentiation. PBMCs from 6 healthy subjects were pretreated with biotinylated Annexin V (biot AnV) (n = 8) or with glyburide (Gly) (n = 8) and then stimulated for 24 hr according to the protocol for B10 assessment previously described. A shows representative plots and B shows the results as median (IQR25-75).

To assess the impact of PS translocation blockage on IL-10 secretion, we incubated PBMCs from 6 healthy subjects with either biotinylated annexin V or glyburide that is an inhibitor of phospholipid translocase interfering with PS exposure. PBMCs were cultured alone or with biotinylated Annexin V or with glyburide for 24 hr according to the protocol for B10 assessment as previously described. The median (IQR25-75) frequency of B10 cells was 5.49 (3.85–7.70)% vs 3.53 (1.94–4.89)% vs 2.65 (1.18–4.26)% for culture medium alone, biotinylated Annexin V and glyburide respectively (Fig 5A and 5B). A lower frequency of B10 cells was observed in the presence of biotinylated Annexin V (p = 0.0078; n = 8) and with glyburide (p = 0.015, n = 8)

## Discussion

This study showed that B10 cells and their precursors expose more often PS on the outer layer membrane than other B cells. B cells exposing PS are viable cells that differentiate more into B10 cells. The blockage of PS inhibits B10 cell generation.

Our results suggest that PS exposure occurs in B cells that are prone to produce IL-10. Indeed, B10 cells externalized more PS than IL-10 non-producing B cells as well as CD5+ and CD24^hi^CD27^+^ B cells, known as precursors for B10 cells. Moreover, AnV+ sorted B cells differentiate more frequently into B10 cells than AnV^neg^ B cells after CpG activation. PS exposure may reflect a B-cell differentiation state since, in mice, AnV+ B cells are preferentially found in CD5^+^ B-1 and splenic marginal zone B cells (14). Moreover, in the present study, PS exposure is more pronounced on CD5^+^ B cells than on other precursors. PS exposure may also reflect an activation state as PS co-caps with IgM, CD19 and MHC class I and cross-linking PS alters BCR signaling [13]. PS exposure also reflects increased intracellular calcium concentrations as previously shown [22]. Indeed, CpG elicits a large calcium elevation in B cells using a TLR9 independent pathway and when the calcium influx is impaired in B cells, IL-10 secretion decreased [23,24].

PS may amplify B cells IL-10 production. Apoptotic cells can stimulate IL-10 production by B cells [25]. Recently, it was shown that T cell Ig domain and mucin domain protein 1 (TIM-1), a PS receptor, is responsible for B10 cell induction by apoptotic cells [26]. Indeed, TIM-1 has been described to characterize a subset of B cells having regulatory functions and secreting IL-10 [27]. Thus, it is possible that PS exposure on B cells leads to TIM-1 activation and IL-10 production of other B cells, acting as an amplification loop. Blockage of PS exposure by glyburide or biotinylated AnV impaired B cell IL-10 secretion. This could be explained by an inhibition of this amplification loop.

Tumors have long been recognized as having immunosuppressive microenvironments that alters the ability of host to control tumor growth. It has recently been shown that exposure of PS contributes to this immunosuppressive state. Indeed, PS is exposed on endothelial cells in the tumor vasculature and on some tumor cells [28,29]. Exposed PS is recognized by macrophages and dendritic cells which engulf the PS-expressing cells and produce IL-10- and TGF-β–dependent immunosuppressive signals [30,31]. Bavituximab, a PS-targeting antibody currently evaluated in clinical trials for cancer, can suppress tumor growth and progression by changing the tumor microenvironment from immunosuppressive to immunostimulatory by converting tumor-associated macrophages from M2 (anti-inflammatory profile) to M1 (proinflammatory profile) [32]. Our work suggests that inhibition of PS by biotinylated AnV or glyburide could also induce a decrease of B10 cells. It is important, however, to remain cautious regarding the mechanism of action of glyburide, also known as glibenclamide, that is a treatment of type 2 diabetes mellitus with an inhibitory effect, not only on PS but also on inflammasome assembly [33]. The pro-inflammatory effect of glyburide may not be exclusively explained by PS blockage.

Phosphatidylserine exposure has been identified as an “eatme signal” for phagocytes [34]. The lack of response to such stimuli towards those B cells might be explained by the absence of soluble mediators, usually released by apoptotic cells to attract phagocytes (“find-me” signals) and most probably by the presence of "don't eat me" signals. This would require further studies.

In conclusion, PS exposure is increased in B10 cells and their precursors. Inhibition of PS decreases B cells differentiation into B10 cells. This could participate to the reactivation of anti-tumor immunity observed with PS-targeting antibodies in oncology. In auto-immune diseases, inhibition of PS flippase to increase PS exposure could be a way to restore regulatory B cells.

## Supporting Information

S1 Fig Gating strategy for flow cytometry analysis. First, PBMCs were gated based on forward scatter (FSC) and side scatter (SSC). Single cells from gate a were further gated on with side scatter height (SSC-H) versus SSC width (SSC-W) and then with FSC-H versus FSC-W. Then, alive B cells were gated according to CD19 positive expression and negative Viability Dye.
(TIF)

S2 Fig Annexin V binding remains increased in IL-10 producing B cells (B10 cells) compared to IL-10 non-producing B cells when using APC-conjugated annexine V. PBMCs were stimulated for 24 hr with CpG/ionomycin/PMA and BFA as described previously and analyzed by FACS for annexin V (AnV) binding and IL-10 in 13 subjects. Dead cells were assessed by their positivity to eFluor 506. Results are presented in percentage of positive cells. Wilcoxon’s matched pairs signed rank tests were used. Top of the bar represents the median, and whiskers are IQR 25–75.
(TIF)

S3 Fig CpG stimulation do not change the AnV staining of B cells. Representative plot of AnV staining with and without CpG stimulation (A) and comparison of the AnV+B cells percentages among B cells stimulated or not stimulated with CpG (n = 6)(B). Wilcoxon’s matched pairs signed rank tests were used. Data are median (IQR25-75).
(TIF)
