# Supplementary figures and images for: Phosphatidylserine Outer Layer Translocation Is Implicated in IL-10 Secretion by Human Regulatory B Cells
Source: PLoS One. 2017 Jan 10;12(1):e0169755. doi: 10.1371/journal.pone.0169755 (PMC5225009; doi:10.1371/journal.pone.0169755)

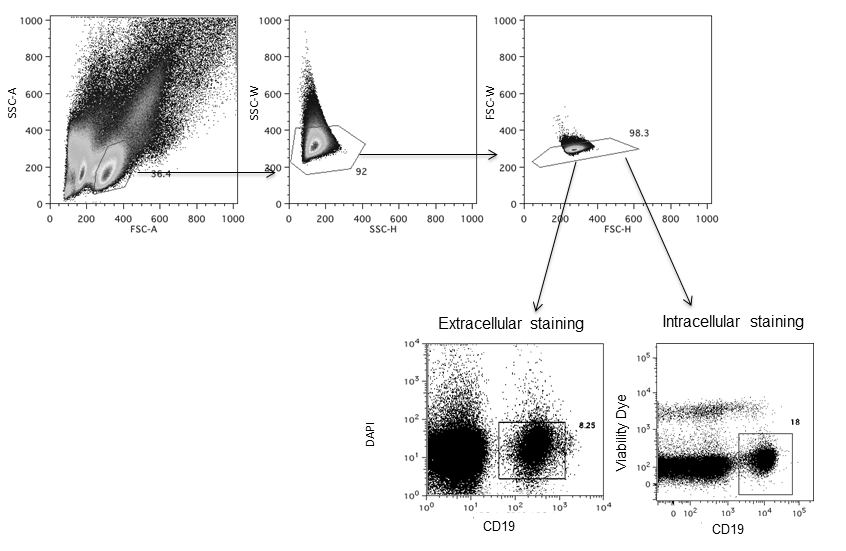

Supplement: S1 Fig — First, PBMCs were gated based on forward scatter (FSC) and side scatter (SSC). Single cells from gate a were further gated on with side scatter height (SSC-H) versus SSC width (SSC-W) and then with FSC-H versus FSC-W. Then, alive B cells were gated according to CD19 positive expression and negative Viability Dye. (TIF) [file pone.0169755.s001.tif]

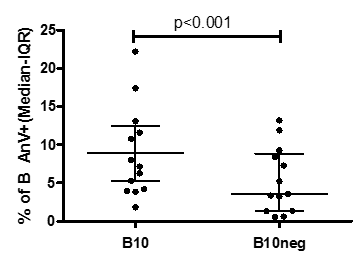

Supplement: S2 Fig — PBMCs were stimulated for 24 hr with CpG/ionomycin/PMA and BFA as described previously and analyzed by FACS for annexin V (AnV) binding and IL-10 in 13 subjects. Dead cells were assessed by their positivity to eFluor 506. Results are presented in percentage of positive cells. Wilcoxon’s matched pairs signed rank tests were used. Top of the bar represents the median, and whiskers are IQR 25–75. (TIF) [file pone.0169755.s002.tif]

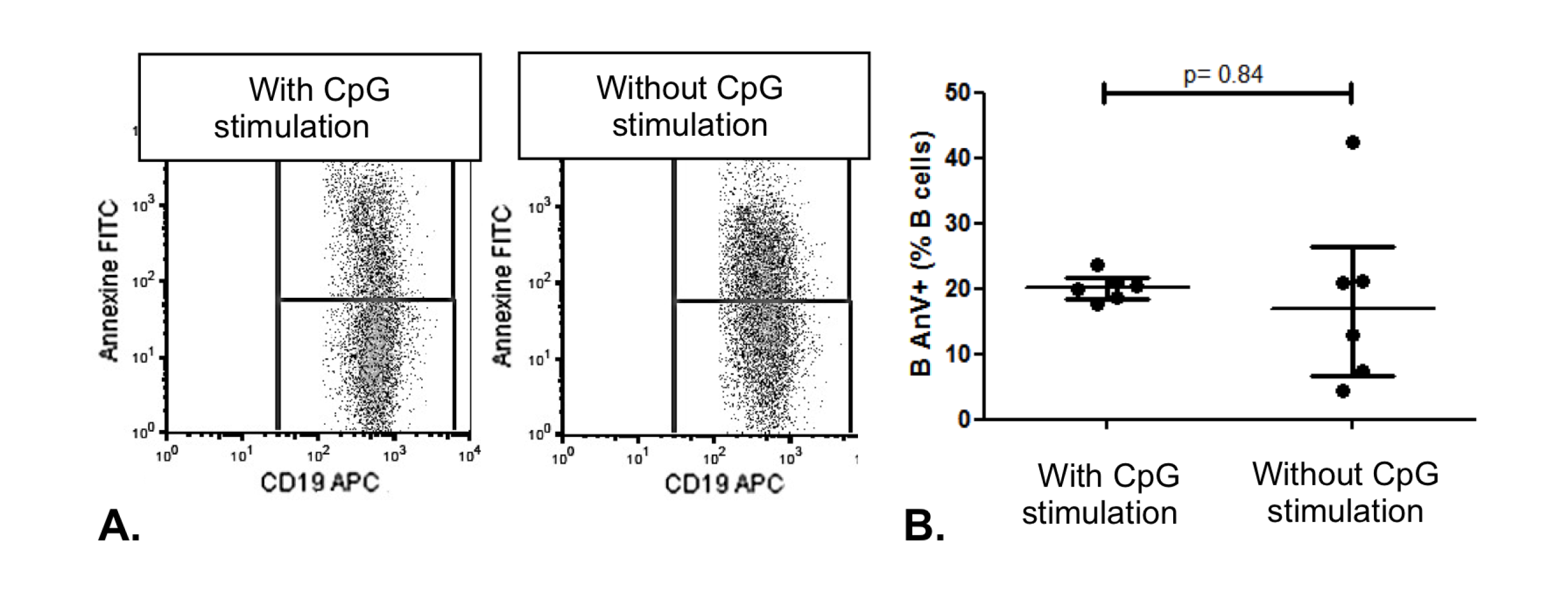

Supplement: S3 Fig — Representative plot of AnV staining with and without CpG stimulation (A) and comparison of the AnV+B cells percentages among B cells stimulated or not stimulated with CpG (n = 6)(B). Wilcoxon’s matched pairs signed rank tests were used. Data are median (IQR25-75). (TIF) [file pone.0169755.s003.tif]
